# Effect of Acute *Plasmodium falciparum* Malaria on Reactivation and Shedding of the Eight Human Herpes Viruses

**DOI:** 10.1371/journal.pone.0026266

**Published:** 2011-10-24

**Authors:** Arnaud Chêne, Susanne Nylén, Daria Donati, Maria Teresa Bejarano, Fred Kironde, Mats Wahlgren, Kerstin I. Falk

**Affiliations:** 1 Department of Microbiology Tumor and Cell Biology (MTC), Karolinska Institutet, Stockholm, Sweden; 2 Department of Medicine, Center for Infectious Medicine, Karolinska Institutet, Stockholm, Sweden; 3 Department of Biochemistry, Faculty of Medicine, Makerere University, Kampala, Uganda; 4 Swedish Institute for Communicable Disease Control, Solna, Sweden; Institut national de la santé et de la recherche médicale - Institut Cochin, France

## Abstract

Human herpes viruses (HHVs) are widely distributed pathogens. In immuno-competent individuals their clinical outcomes are generally benign but in immuno-compromised hosts, primary infection or extensive viral reactivation can lead to critical diseases. *Plasmodium falciparum* malaria profoundly affects the host immune system. In this retrospective study, we evaluated the direct effect of acute *P. falciparum* infection on reactivation and shedding of all known human herpes viruses (HSV-1, HSV-2, VZV, EBV, CMV, HHV-6, HHV-7, HHV-8). We monitored their presence by real time PCR in plasma and saliva of Ugandan children with malaria at the day of admission to the hospital (day-0) and 14 days later (after treatment), or in children with mild infections unrelated to malaria. For each child screened in this study, at least one type of HHV was detected in the saliva. HHV-7 and HHV-6 were detected in more than 70% of the samples and CMV in approximately half. HSV-1, HSV-2, VZV and HHV-8 were detected at lower frequency. During salivary shedding the highest mean viral load was observed for HSV-1 followed by EBV, HHV-7, HHV-6, CMV and HHV-8. After anti-malarial treatment the salivary HSV-1 levels were profoundly diminished or totally cleared. Similarly, four children with malaria had high levels of circulating EBV at day-0, levels that were cleared after anti-malarial treatment confirming the association between *P. falciparum* infection and EBV reactivation. This study shows that acute *P. falciparum* infection can contribute to EBV reactivation in the blood and HSV-1 reactivation in the oral cavity. Taken together our results call for further studies investigating the potential clinical implications of HHVs reactivation in children suffering from malaria.

## Introduction

Human herpes viruses (HHVs) are widely distributed pathogens. Serological evidence of HHV infections are found in the majority of the world's population [1], [2], [3], [4], [5]. Following primary infection, HHVs establish a long life persistent infection within the human host characterized by frequent mild viral reactivation to allow shedding and transmission. The latency tropism of select HHVs is not yet fully characterized. In immuno-competent individuals the clinical outcomes of HHVs are generally benign whereas in immuno-compromised hosts primary infection or viral reactivation can lead to critical diseases such as encephalitis [6], meningitis [7], pneumonitis [8] and to the emergence of malignant disorders [5], [9], [10], [11]. These facts reflect a delicate equilibrium between viral lytic replication and host immune responses. If this balance is upset, failure to restrain the viral load can lead to severe pathogenesis.

Saliva appears to be an important compartment for transmission of several HHVs and it is likely that both the amount and frequency of HHVs in saliva are determinants of risk for transmission [12]. Herpes simplex virus 1 and 2 (HSV-1, HSV-2), varicella-zoster virus (VZV), Epstein-Barr virus (EBV), cytomegalovirus (CMV), and human herpesvirus 6, 7 and 8 (HHV-6, HHV-7, HHV-8) belong to the *Herpesviridae* family commonly infecting people and can be classified in three different sub-families sharing genetic and biological similarities (α, β and γ *herpesviridae*). In affluent countries primary infections by β herpes viruses (CMV, HHV-6, and HHV-7) occur very early in childhood [13], [14] while primary infections by α viruses (HSV-1, HSV-2, VZV) and γ viruses (EBV and HHV-8) are usually delayed until the young adult life. In Sub-Saharan Africa, where malaria is holo-endemic, HHVs primary infections occur earlier in life and herpes viruses are widespread among young children.

The impact of acute malaria infection on HHV reactivation and shedding has not been fully characterized. If α viruses are neurotropic, β and γ viruses can establish latency in certain types of peripheral blood mononuclear cells (PBMCs) and/or in vascular endothelial cells. [15], [16], [17]. Our previous studies indicate that malaria affects EBV persistence as reflected by an increased viral replication. Children living in malaria-endemic areas have elevated EBV loads in the circulation [18], [19] and acute malaria infection leads to increased levels of circulating EBV that are cleared following anti-malaria treatment [20]. Furthermore, we demonstrated that a malarial antigen was able to directly drive an infected B cell into EBV lytic replication [21]. Increased reactivation of the oncogenic EBV could lead to an augmented risk of developing malignancies [22]. Acute *P. falciparum* malaria infection has also been suggested to induce HSV-1 and VZV reactivation [23], [24], [25]. To extend our previous studies and evaluate the direct effect of acute malaria infection on reactivation and shedding of a wider range of HHVs, we analysed the viral DNA load of HSV-1, HSV-2, VZV, EBV, CMV, HHV-6, HHV-7 and HHV-8 in plasma and saliva from children with acute malaria and 14 days after they received anti-malarial treatment.

## Materials and Methods

### Patients

This retrospective study was carried out on samples collected in Kampala, Uganda from October to December, after the second rainy season 2002. Children with malaria and children with mild infections unrelated to malaria were recruited at the Assessment Center, Mulago National Referral Hospital, Kampala, Uganda. Plasma and saliva were collected from children with acute uncomplicated malaria (M^+^) on the day of recruitment (M^+^ day-0, n = 23), from the same children 2 weeks after they had received anti-malaria treatment (M^+^ day-14; n = 23; age range 2–15 years; mean age 6,6 years) and from children with mild infections unrelated to malaria (M^−^; n = 24; age range 2–14 years; mean age 6,8 years). Children admitted to the hospital and presenting *P. falciparum* parasitemia (parasites were detected in their blood smears) were classified in the acute malaria group. At enrolment, M^+^ children received anti-malaria treatment consisting of sulfadoxine (25 mg/kg) and pyrimethamine (1.25 mg/kg) (Fansidar; Roche) on day-0 plus amodiaquine (Camoquine; Park-Davis) (25 mg/kg in 3 divided doses) on days 0, 1, and 2. Some children received paracetamol and/or an anticough mixture. The M^−^ control group consisted of children who visited the clinic with a variety of symptoms unrelated to malaria (no parasites were detected in their blood smears). The study protocols were approved by the ethics committees of Makerere University Faculty of Medicine, the Uganda National Council of Science and Technology, and the Karolinska Institutet. Written, informed consent was obtained from guardians of study participants. Detailed clinical data of the patients are presented in Table S1.

### Blood and saliva samples

Venous blood (2–5 mL) was collected in tubes containing EDTA and was centrifuged to obtain the plasma fraction. Saliva was obtained by the following method: children chewed a sponge for 1 min, the sponge was then washed in 1 mL of PBS. All samples were stored at −20°C until further processing.

### Viral DNA quantification

DNA extraction from plasma and saliva samples was performed using a fully automated workstation (BioRobot® M48; Qiagen, MagAttract® DNA Mini M48 Kit; Qiagen) to insure maximum reliability. Following manufacturer's instructions, DNA was extracted from 200 µL of samples (plasma or saliva) and eluted in 200 µL DNAse free water.

Viral DNA quantification of HSV-1, HSV-2, VZV, EBV, CMV, HHV-6, HHV-7 and HHV-8 was assessed by real time polymerase chain reaction (PCR) according to optimized protocols [26] approved and used for routine diagnostics at the Swedish Institute for Communicable Disease Control, Solna, Sweden. The PCR was carried out in 96-well plates in a final volume of 25 µL including 5 µL of DNA sample. Briefly, the PCR mixture consisted of 12,5 µL of TaqMan universal PCR mix (Applied Biosystems) and various amounts and concentrations of MgCl_2_, water, primers and fluorescence-labelled probes. Cycling parameters were 50°C for 2 min, 95°C for 10 min, 45 cycles 95°C for 15 s, and 60°C for 1 min. Detection was performed using an ABI Prism 7900 Sequence Detection System (Applied Biosystems). Data were analyzed with SDS2.3 software (Applied Biosystems) in reference to a standard curve prepared using serial dilutions of DNA derived from the EBV-positive BL line Namalwa that contains two copies of EBV genome per cell. The sensitivity of the assay (detection limit) was 1–5 viral copies per reaction well. As positive control, DNA of a select HHV (50-100 copies/mL) was added to the assay to ensure PCR reliability. The DNA copy number for each virus was calculated as the mean value of triplicates.

### Statistical analysis

Given the small sample size, statistical analyses were performed using non-parametric statistical tests. Wilcoxon Signed-Ranks Test for paired analysis and Mann-Whitney Rank-Sum Test for group comparison were used. P<0.05 was considered to be statistically significant.

## Results

We determined the prevalence (detection rate) and loads of HSV-1, HSV-2, VZV, EBV, CMV, HHV-6, HHV-7 and HHV-8 regardless of the patient HHVs serological status in plasma and saliva samples from children having acute *P. falciparum* infection at the date of admission at the hospital (M^+^ day-0), 14 days after anti-malarial treatment (M^+^ day-14). The M^-^ group, composed of Ugandan children that are age-, sex-matched and of same ethnical and geographical origins to the M^+^ group is used to ascertain that any effect on viral reactivation and/or shedding observed within the M^+^ group between day-0 and day-14 would most exclusively result from malaria infection and not from other types of illness.

### Prevalence of HHVs in saliva and plasma

**Figure 1:**
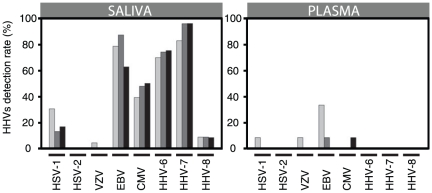
Prevalence of HHVs in saliva and plasma. Detection rate of HHVs in saliva samples and plasma samples from children with acute malaria before (M^+^ day-0; light gray bars) and after (M^+^ day-14; dark gray bars) anti-malarial treatment and in children having mild infections unrelated to malaria (M^−^; black bars). All samples were considered for this analysis, regardless the serological status of the patients for a select HHV.

For each child screened in this study, at least one type of HHV was detected in the saliva. [Table S2]. The order of prevalence of the tested viruses was the same in the salivary samples from M^+^ at day-0 and at day-14. HHV-7, EBV and HHV-6 were detected in more than two third of the samples, CMV in almost half of the samples. HSV-1, HHV-8, and VZV were detected at lower frequency. It is noticeable that the proportion of saliva samples with detectable HSV-1 was much lower after anti-malaria treatment (30% at day-0 and 13% at day-14) [Figure 1] even though this difference was not statistically significant due to the limited number of samples.

The HHVs detection rate was lower in the plasma that in the saliva samples. Forty two per cent (5/12) of the plasma samples M^+^ day-0 and 8% (1/12) of the plasma samples from the M^+^ day-14 had detectable viral DNA levels of at least one HHV. At day-0 the most prevalent virus in the plasma was EBV (33%) followed by HSV-1 and VZV (8%) while at day-14 only 8% (1/12) of the samples had detectable EBV.

In the M^−^ group, only one child had detectable HHV in the plasma (patient 36 positive for CMV). Salivary HHVs prevalence was as follow; HHV-7 (96%), HHV-6 (75%), EBV (63%), CMV (50%), HSV-1 (17%), HHV-8 (8%), VZV (0%) and HSV-2 (0%).

HSV-2 was not detected in any of the samples regardless the group or the physiological compartment tested.

### HHVs loads in saliva and plasma

#### α herpes viruses

**Figure 2:**
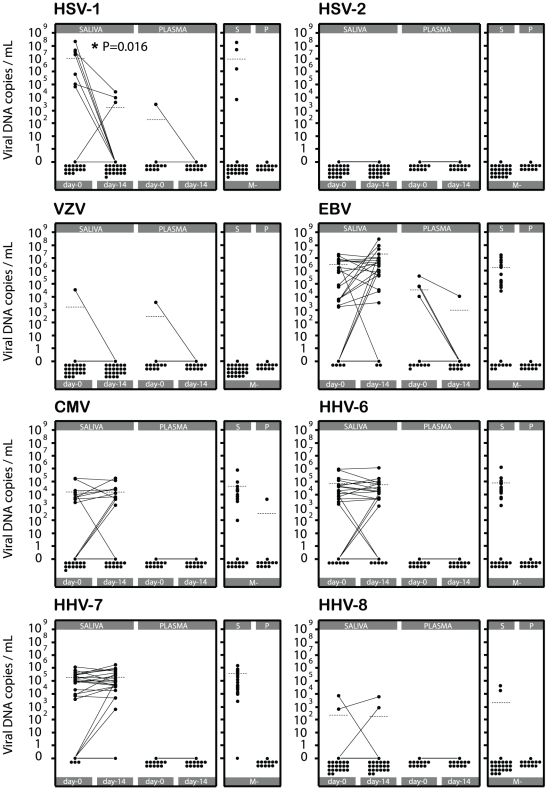
HHVs loads in saliva and plasma before and after anti-malarial treatment. HHVs loads (viral DNA copies/mL) in saliva and plasma samples from children with acute malaria before (day-0) and after (day-14) anti-malarial treatment and in children having mild infections unrelated to malaria (M^−^). Each black circle represents the viral load for a select HHV for one patient. The black continuous lines link the viral loads of a select HHV at day-0 and at day-14 for the same patient. Paired analysis between the M^+^ day-0 and M^+^ day-14 samples were performed using the Wilcoxon Signed-Ranks Test. P<0.05 was considered to be statistically significant. The dashed horizontal lines represent the mean viral load for a select HHV in each group. All samples with less than 200 viral DNA copies/mL were considered negative.

Paired analysis of HSV-1 levels for each patient between day-0 and day-14 showed a profound reduction of HSV-1 levels in the saliva after anti-malarial treatment (Wilcoxon Signed-Ranks Test; P = 0.016) [Figure 2]. One child (1/12) had detectable HSV-1 in both the plasma (2.9×10^3^ copies/mL) and the saliva (3.4×10^7^ copies/mL) at day-0. HSV-1 was not detected in the plasma of the same patient after anti-malarial treatment while the HSV-1 viral load in the saliva was profoundly reduced (2.7×10^4^ copies/mL). Group comparison using the Mann-Whitley Rank-Sum Test between M^+^ day-0 and M^−^ saliva samples showed that the respective levels of HSV-1 were not significantly different (P = 0.289). HSV-1 was not detectable in any of the M^−^ plasma samples.

HSV-2 was not detectable in any of the samples of the M^+^ or M^−^ groups whereas one patient had detectable levels of VZV in the saliva (3.4×10^4^ copies/mL) and in the plasma (3.6×10^3^ copies/mL) at day-0. VZV was cleared from the saliva and the plasma after the patient received anti-malarial treatment. VZV was not detectable in any of the M^−^ samples.

#### β herpes viruses

Paired analysis of CMV levels for each patient between day-0 and day-14 showed that the respective levels of CMV in the saliva were not significantly affected (Wilcoxon Signed-Ranks Test; P = 0.424) [Figure 2]. CMV was not detectable in any of the plasma samples M^+^. The mean salivary CMV load in the M^−^ group was 4.1×10^4^ copies/mL. Group comparison using the Mann-Whitley Rank-Sum Test between M^+^ day-0 and M^−^ saliva samples showed that the respective levels of CMV were not significantly different (P = 0.407). Only one plasma sample (1/12) had a detectable level of CMV (4.4×10^3^ copies/mL).

Paired analysis of HHV-6 levels for each patient between day-0 and day-14 showed that the respective levels of HHV-6 in the saliva were not significantly affected (Wilcoxon Signed-Ranks Test; P = 0.953). HHV-6 was not detectable in any of the plasma samples of the M^+^ or M^−^ groups. Group comparison using the Mann-Whitley Rank-Sum Test between M^+^ day-0 and M^-^ saliva samples showed that the respective levels of HHV-6 were not significantly different (P = 0.519).

Paired analysis of HHV-7 levels for each patient between day-0 and day-14 showed that the respective levels of HHV-7 in the saliva were not significantly affected (Wilcoxon Signed-Ranks Test; P = 0.270). HHV-7 was not detectable in any of the plasma samples of the M^+^ or M^−^ groups. Group comparison using the Mann-Whitley Rank-Sum Test between M^+^ day-0 and M^-^ saliva samples showed that the respective levels of HHV-7 were not significantly different (P = 0.327).

#### γ herpes viruses

Paired analysis of EBV levels for each patient between day-0 and day-14 showed that the respective levels of EBV in the saliva were not significantly affected (Wilcoxon Signed-Ranks Test; P = 0.211) [Figure 2]. Four plasma samples (4/12) showed detectable levels of EBV (mean 4.5×10^4^ copies/mL) at day-0. After anti-malarial, EBV was no longer detectable in 3 of these patients and had dropped from 4×10^5^ copies/mL to 1×10^4^ copies/mL for the fourth one. Due to the small number of samples this trend is not statistically significant (Wilcoxon Signed-Ranks Test; P = 0.125) but is in line with previously published results performed at a larger scale [20]. Group comparison using the Mann-Whitley Rank-Sum Test between M^+^ day-0 and M^-^ salivary samples showed that the respective levels of EBV were not significantly different (P = 0.575). EBV was not detectable in any of the M^−^ plasma samples.

HHV8 was detected in only two salivary samples from each group.

### HHVs loads in saliva during viral shedding

In order to assess the HHVs loads in saliva during shedding among the 3 groups we determined the mean viral DNA copy numbers in salivary samples for each herpes virus. Only samples with detectable viral DNA, reflecting viral shedding, were considered for this analysis [Table S2].

#### α herpes viruses

**Figure 3:**
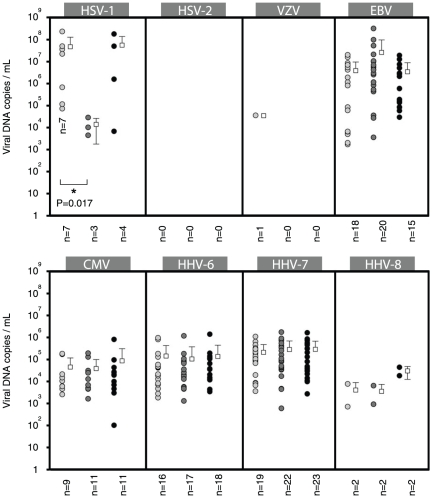
Salivary shedding of HHVs before and after anti-malarial treatment. Quantitative comparison of HHVs loads (viral DNA copies/mL) during salivary shedding in children with acute malaria before (M^+^ day-0; light gray circles) and after (M^+^ day-14; dark gray circles) anti-malarial treatment and in children having mild infections unrelated to malaria (M^−^; black circles). Each white circle represents the viral load for a select HHV for one patient. White squares depict the mean values, with standard deviations. For a select HHV, only samples with detectable viral DNA, reflecting salivary shedding, were considered for this analysis (n = number of samples analyzed).

Group comparison showed a profound reduction of HSV-1 DNA levels in the saliva after anti-malarial treatment (Mann-Whitney Rank-Sum Test; P = 0.017) [Figure 3]. The mean HSV-1 load in the saliva samples from the M- group (5.5×10^7^ copies/mL) was similar to the mean viral load in the M^+^ day-0 saliva samples. No viral DNA was detectable in any of the saliva samples for HSV-2 and only one patient (1/23) had detectable VZV in the saliva at day-0 (3.4×10^4^ copies/mL).

#### β herpes viruses

Group comparison of each viral load using the Mann-Whitney Rank-Sum Test between M^+^ day-0, M^+^ day-14 and M^−^ saliva samples showed that the respective levels of β herpes viruses were not significantly different [Figure 3].

#### γ herpes viruses

Group comparison of EBV loads using the Mann-Whitney Rank-Sum Test between M^+^ day-0, M^+^ day-14 and M^−^ saliva samples showed that the respective levels of EBV were not significantly different [Figure 3]. Despite the few numbers of saliva samples with detectable HHV-8 (2 in each group), the calculated means of salivary HHV-8 copies in saliva samples M^+^ day-0, M^+^ day-14 and M^-^ were respectively 3.9×10^3^, 3.4×10^3^ and 2.9×10^4^ copies/mL.

**Figure 4:**
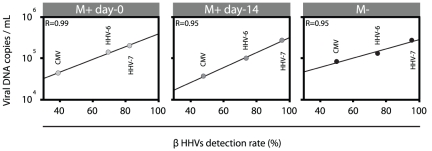
β herpes viruses prevalence and shedding. Correlation established by linear regression analysis between β herpes viruses loads during salivary shedding (data from Figure 3) and β herpes viruses prevalence (detection rate) in all the saliva sample analyzed. (M^+^ day-0; light gray circles), (M^+^ day-14; dark gray circles), (M^−^; black circles).

During salivary shedding in the M^+^ day-0 and M^−^ groups, the highest mean viral load was observed for HSV-1 followed by EBV, HHV-7, HHV-6, CMV and HHV-8. In the M^+^ day-14 group there was a drastic reduction of HSV-1 levels in the saliva, the highest salivary viral load being observed for EBV followed by HHV-7, HHV-6, CMV, HHV-8 and HSV-1. In the 3 groups, linear regression analysis could establish a correlation between β herpes virus DNA levels during salivary shedding and prevalence of virus detection in the saliva (M^+^ day-0, R = 0.99; M+ day-14, R = 0.95; M-, R = 095). During shedding, an increased viral load for a select β herpes virus was correlated to a higher detection rate [Figure 4]. The efficiency of β herpes viruses transmission within a population is most likely directly proportional to the number of viral particles present in the saliva during shedding confirming that saliva is a privileged route of transmission for CMV, HHV-6 and HHV-7.

## Discussion

HHVs infections are usually benign in immuno-competent individuals. Nevertheless, extensive viral reactivation under certain conditions such as immuno-suppression can lead to severe illness. Detection of herpes virus in plasma of patients with AIDS or after stem cell transplantation has been widely reported [27], [28]. Monitoring herpes viruses activity by viral quantification in plasma or saliva samples can serve as a useful diagnostic tool for viral associated diseases [29], [30].

*P. falciparum* malaria profoundly affects the host immune system by mediating at the same time immuno-suppression and immune hyper-activation. This is reflected by the impairment of macrophage function and antigen presentation (dendritic cell inhibition), by a diminished specific T-cell response and the induction of regulatory T cells. Polyclonal B cell activation leads to hyper-gamma-immunoglobulinemia and production of auto-antibodies. *P. falciparum* infection is also accompanied by high plasma levels of pro-inflammatory cytokines (IL-6, TNFα) and regulatory cytokines (IL-10, TGFβ) [31]. This immunological state could be envisaged as favorable for the reactivation of latent herpes viruses. Furthermore, direct contacts between malarial antigens and latently infected cells may also result in viral reactivation as we have previously described for a PfEMP1-CIDR1α variant that is able to drive an EBV latently infected B cell into lytic replication [21]. In this report, we have screened all known human herpes viruses in plasma and saliva samples from children with acute uncomplicated *P. falciparum* infection and followed up the same patients 14 days after anti-malarial treatment.

Neurotropic *α* herpes viruses (HSV-1, HSV-2, VZV) establish latent infection mainly in the trigeminal and dorsal root ganglia maintaining the potential to resume viral replication to insure shedding and transmission. In immuno-competent individuals, VZV reactivation occurs mainly once in the lifetime while it is recurrent for HSV-1 and HSV-2 generally associated with mild to moderate illness. Our results show that some children having acute *P. falciparum* carry high levels of HSV-1 in the saliva. After anti-malarial treatment the salivary HSV-1 levels were profoundly diminished and totally cleared in 5 out of 7 positive children. In the M^+^ group, one plasma sample had detectable of HSV-1 at day-0, the virus been cleared at day-14. The results are in line with previous studies associating clinical manifestations of herpes labialis (HSV-1) to acute *P. falciparum* in children [23]. A variety of factors such as stress and fever have been shown to be able to induce reactivation of herpes simplex viruses. HSV-1 was also detected in the saliva samples from the malaria negative study group. In the M- group HSV-1 reactivation in the oral cavity could be attributed to the fact that these patients have mild infections, active ongoing immune response and associated fever (Table S1). Nevertheless, the detection rate of HSV-1 in the saliva samples from the M^+^ day-0 group (7/23) was almost double that of the M^−^ group (4/24). The observed decrease in HSV-1 levels in the saliva between day-0 and day-14 is unlikely to result from a potential antiviral property of the treatment since the salivary levels of the other HHVs were not affected.

HSV-2, thought to be mainly sexually transmitted, was not detected in any of the children assessed. One child with acute malaria had noticeable VZV in the saliva and in the plasma the day of admission to the hospital. After anti-malaria treatment VZV was no longer detectable either in the saliva or in the plasma and a number of case reports indicate that herpes zoster reactivation can occur during *P. falciparum* [24], [25], [32].

Reactivation of α herpes viruses can lead to severe neurological disorders such as herpes simplex encephalitis (HSE) or acute disseminated encephalomyelitis (ADEM) [6], [7], [32]. The data presented here together with previous reports on reactivation of *α* herpes viruses in malaria, call for studies investigating if *α* herpes viruses may contribute to neurological complications, commonly diagnosed as cerebral malaria, during *P. falciparum* infection. Saliva appears to be a relevant compartment to assess *α* herpes viruses reactivation. Furthermore, measuring HSV loads in the saliva rather that in the plasma could present substantial advantages for routine screenings since this compartment is more accessible. Nevertheless, even if monitoring neurotropic viruses in other compartments, like the cerebrospinal fluids, might also be of interest access to such samples could prove difficult.

Beta herpes viruses (CMV, HHV-6 and HHV-7) can establish latent infections in peripheral mononuclear cells (PBMCs) [16], [17] and can be reactivated in immuno-suppressed individuals and lead to clinical symptoms [27], [33]. *P. falciparum* infected erythrocytes have been reported to directly interact with a variety of PBMCs such as monocytes/macrophages, platelets, granulocytes [34], dendritic cells [35] and B cells [36]. It was thus of interest to investigate if these interactions could lead to viral reactivation. Moreover, HHV-6 and HHV-7 are considered to be causative agents of exanthema subitum that can lead to febrile seizures. Several reports also suggest that HHV-6 and HHV-7 can invade the central nervous system and lead to severe neurological manifestations [37], [38], [39]. In our study, we observed no significant change in β herpes viral load between the M^+^ day-0, M^+^ day-14 and the M^-^ saliva samples. Beta herpes viruses were not detected in any of the plasma samples from the M^+^ groups.

The association between malaria and EBV reactivation has already been established and the data has been thoroughly discussed elsewhere [18], [20], [22]. HHV-8 belongs to the same γ herpes virus sub-family as EBV, sharing genetic and biological similarities. As EBV, HHV-8 can establish latency in B cells and is also linked to malignancies. [10]. These similarities with EBV make assessment of HHV-8 reactivation interesting in malaria patients. Moreover, epidemiological studies from Italy revealed a drop in HHV-8 sero-prevalence over time coinciding with the drop of malaria vectors in the same geographical setting [40], [41]. In the samples assessed here, the incidence of HHV-8 was too low to draw any conclusions since only 2/23 patients in each group had detectable HHV-8 in the saliva.

We conclude from our study that acute *P falciparum* infections can contribute to EBV reactivation in the blood and HSV-1 reactivation in the oral cavity. Acute malaria was not associated with reactivation of the β herpes viruses CMV, HHV-6 and HHV-7 and the sample size in this study was too small to draw any conclusions on the impact of malaria on VZV and HHV-8 reactivation. A prospective study including a larger number of individuals would be required. The age group examined was probably too young for assessment of HSV-2 reactivation. Taken together our results call for further studies investigating the potential clinical implications of HHVs reactivation in children suffering from malaria.

## Supporting Information

Table S1 **Clinical characteristics of the patients.** Clinical characteristics of the patients belonging to the M^+^ group and the M^−^ group. “−“indicates that the data are missing or not determined.
(DOC)

Table S2 **HHVs loads in saliva and plasma.** HHVs load (viral DNA copies/mL) in plasma samples (P) and saliva (S) from children with acute malaria before and after anti-malarial treatment and in children having mild infections unrelated to malaria. All samples with less than 200 viral copies/mL were considered negative (0). “−“indicates that the data are not determined.
(DOC)
