# Supplementary material for: Effect of Acute Plasmodium falciparum Malaria on Reactivation and Shedding of the Eight Human Herpes Viruses
Source: PLoS One. 2011 Oct 24;6(10):e26266. doi: 10.1371/journal.pone.0026266 (PMC3200318; doi:10.1371/journal.pone.0026266)
Supplement: Table S1 — Clinical characteristics of the patients. Clinical characteristics of the patients belonging to the M+ group and the M− group. “−“indicates that the data are missing or not determined. (DOC) [file pone.0026266.s001.doc]

**Table S1**

|  | | | | **Clinical symptoms (day-0 / day-14)** | | | | | | | | | **Parasitemia** | |
| --- | --- | --- | --- | --- | --- | --- | --- | --- | --- | --- | --- | --- | --- | --- |
| **Patients** | **Age (y)** | **Sex** | **Weight**  **(Kg)** | **Fever** | **Anorexia** | **Headache** | **Vomiting** | **Rash** | **Diarrhea** | **Cough** | **Jaundice** | **Abdominal Pain** | ***Pf* / µL**  **day-0** | ***Pf* / µL**  **day-14** |
| **1** | 4 | M | 13 | Y / N | Y / N | Y / N | N / N | Y / N | N / N | N / Y | N / N | N / N | 43 520 | 0 |
| **2** | 2,5 | F | 12 | Y / N | N / N | - / - | N / N | Y / N | N / N | Y / Y | N / N | - / - | 26 180 | 1 440 |
| **3** | 4 | F | 11 | N / N | Y / N | N / N | Y / N | N / Y | N / N | N / Y | N / N | N / N | 69 880 | 7 480 |
| **4** | 8 | F | 25 | Y / Y | Y / Y | Y / Y | N / N | N / N | N / Y | Y / Y | N / N | N / Y | 30 820 | 0 |
| **5** | 6 | F | 15 | Y / Y | Y / N | N / N | Y / N | N / N | N / N | Y / Y | N / N | N / N | 177 320 | 0 |
| **6** | 12 | M | 39 | Y / N | Y / N | N / N | N / N | - / - | N / N | N / N | N / N | N / N | 35 500 | 40 |
| **7** | 2 | F | 10 | Y / Y | Y / Y | - / - | Y / Y | N / N | N / N | Y / Y | N / N | N / N | 31 000 | 0 |
| **8** | 15 | F | 41 | Y / N | N / N | Y / N | Y / N | N / N | N / N | N / N | - / N | N / N | 73 440 | 0 |
| **9** | 11 | F | 30 | Y / N | N / N | Y / N | N / N | N / N | N / N | Y / N | N / N | Y / - | 11 200 | 0 |
| **10** | 9 | F | 22 | Y / N | N / N | Y / N | N / N | N / N | N / N | N / N | N / N | Y / Y | 18 680 | 0 |
| **11** | 6 | M | 18 | Y / N | N / N | Y / N | N / N | Y / N | N / N | N / N | Y / N | Y / N | 20 000 | 0 |
| **12** | 10 | M | 32 | Y / N | N / N | N / N | Y / N | Y / N | N / N | N / N | Y / N | Y / Y | 20 180 | 0 |
| **13** | 4,5 | M | 13 | Y / N | N / N | Y / N | Y / N | N / N | N / N | Y / N | N / N | N / N | 39 720 | 0 |
| **14** | 3,5 | M | - | Y / N | Y / Y | Y / N | Y / N | N / Y | Y / N | Y / Y | N / N | Y / N | 32 000 | 0 |
| **15** | 2,5 | F | 11 | Y / N | Y / N | - / N | N / N | Y / N | N / N | Y / Y | N / N | - / - | 1 680 | 0 |
| **16** | 4 | F | 15 | Y / N | N / N | Y / N | Y / N | N / N | N / N | Y / N | N / N | N / N | 22 720 | 0 |
| **17** | 8 | M | 24 | Y / N | N / N | N / N | N / N | N / N | N / N | Y / N | N / N | N / N | 200 440 | 0 |
| **18** | 9 | F | 19 | Y / N | Y / N | Y / N | Y / N | N / N | N / N | Y / Y | N / N | Y / N | 280 000 | 0 |
| **19** | 11 | F | 25 | Y / N | N / N | Y / N | Y / N | N / Y | N / N | Y / N | N / N | N / Y | 59 600 | 0 |
| **20** | 2 | F | 14 | Y / N | Y / Y | Y / N | Y / N | N / N | Y / N | Y / Y | N / N | Y / N | 69 090 | 0 |
| **21** | 3 | M | 13 | Y / N | Y / N | Y / N | N / N | N / N | N / N | Y / Y | N / N | N / N | 16 980 | 0 |
| **22** | 4 | M | 17 | Y / Y | N / N | N / N | Y / N | N / N | N / N | Y / Y | N / N | Y / Y | 17 280 | 0 |
| **23** | 12 | F | 23 | Y / N | Y / N | Y / N | N / N | N / N | N / N | N / N | N / N | N / N | 49 280 | 0 |

**M^+^ group**

**M^-^ group**

|  | | | | **Clinical symptoms** | | | | | | | | |
| --- | --- | --- | --- | --- | --- | --- | --- | --- | --- | --- | --- | --- |
| **Patients** | **Age (y)** | **Sex** | **Weight**  **(Kg)** | **Fever** | **Anorexia** | **Headache** | **Vomiting** | **Rash** | **Diarrhea** | **Cough** | **Jaundice** | **Abdominal Pain** |
| **24** | 8 | M | 31 | Y | Y | Y | N | N | - | Y | N | N |
| **25** | 6 | M | 20 | Y | Y | Y | N | N | N | Y | N | Y |
| **26** | 8 | F | 24 | Y | N | - | Y | N | N | Y | N | - |
| **27** | 10 | F | 30 | Y | Y | Y | Y | N | N | Y | N | Y |
| **28** | 8 | F | 21 | Y | N | N | N | N | N | N | N | Y |
| **29** | 12 | M | 40 | Y | Y | Y | N | N | Y | N | N | N |
| **30** | 6 | F | 17 | Y | Y | Y | N | N | N | Y | N | Y |
| **31** | 5 | M | 15 | Y | Y | N | N | N | N | Y | N | N |
| **32** | 7 | M | 24 | Y | N | N | N | N | N | Y | N | N |
| **33** | 5 | M | - | Y | Y | N | N | N | N | Y | N | N |
| **34** | 9,5 | M | 27 | Y | N | Y | N | N | N | Y | N | Y |
| **35** | 4 | F | 14 | Y | Y | Y | N | N | N | Y | N | N |
| **36** | 4 | M | - | Y | N | Y | N | Y | Y | Y | N | Y |
| **37** | 4 | F | 16 | Y | N | N | N | N | N | Y | N | Y |
| **38** | 3 | M | 12 | Y | Y | Y | N | N | N | N | Y | Y |
| **39** | 2 | F | - | Y | N | - | N | N | N | Y | - | - |
| **40** | 13 | M | 34 | Y | N | Y | N | N | N | Y | N | Y |
| **41** | 4 | F | 13 | Y | N | N | N | Y | N | Y | N | N |
| **42** | 4 | M | - | Y | Y | N | Y | Y | Y | Y | N | Y |
| **43** | 8 | F | 18 | Y | Y | N | N | Y | N | Y | N | N |
| **44** | 14 | F | 50 | Y | N | Y | Y | N | N | N | N | N |
| **45** | 12 | M | 25 | Y | Y | Y | N | N | N | Y | N | Y |
| **46** | 6 | F | 22 | Y | N | N | N | N | N | Y | N | N |
| **47** | 2,5 | M | 11 | Y | N | - | N | N | N | Y | N | N |
