# Supplementary material for: Effect of Acute Plasmodium falciparum Malaria on Reactivation and Shedding of the Eight Human Herpes Viruses
Source: PLoS One. 2011 Oct 24;6(10):e26266. doi: 10.1371/journal.pone.0026266 (PMC3200318; doi:10.1371/journal.pone.0026266)
Supplement: Table S2 — HHVs loads in saliva and plasma. HHVs load (viral DNA copies/mL) in plasma samples (P) and saliva (S) from children with acute malaria before and after anti-malarial treatment and in children having mild infections unrelated to malaria. All samples with less than 200 viral copies/mL were considered negative (0). “−“indicates that the data are not determined. (DOC) [file pone.0026266.s002.doc]

**Table S2**

**M^+^ day-0 group**

|  | | | | | | | | | | | | | | | | |
| --- | --- | --- | --- | --- | --- | --- | --- | --- | --- | --- | --- | --- | --- | --- | --- | --- |
|  | **HSV-1** | | **HSV-2** | | **VZV** | | **EBV** | | **CMV** | | **HHV-6** | | **HHV-7** | | **HHV-8** | |
| **Pt.** | **P** | **S** | **P** | **S** | **P** | **S** | **P** | **S** | **P** | **S** | **P** | **S** | **P** | **S** | **P** | **S** |
| 01 | 0 | 0 | 0 | 0 | 0 | 0 | 0 | 1.1x10^6^ | 0 | 0 | 0 | 7.6x10^5^ | 0 | 5.9x10^5^ | 0 | 0 |
| 02 | 0 | 0 | 0 | 0 | 0 | 0 | 0 | 1.8x10^6^ | 0 | 9.8x10^3^ | 0 | 0 | 0 | 4.2x10^5^ | 0 | 0 |
| 03 | 0 | 0 | 0 | 0 | 3.6x10^3^ | 3.4x10^4^ | 0 | 1.6x10^3^ | 0 | 0 | 0 | 3.9x10^4^ | 0 | 1.1x10^5^ | 0 | 6.6x10^2^ |
| 04 | 0 | 0 | 0 | 0 | 0 | 0 | 0 | 4.0x10^6^ | 0 | 0 | 0 | 1.7x10^3^ | 0 | 0 | 0 | 0 |
| 05 | 0 | 2.2x10^8^ | 0 | 0 | 0 | 0 | 0 | 8.0x10^4^ | 0 | 1.4x10^4^ | 0 | 4.1x10^4^ | 0 | 3.3x10^3^ | 0 | 0 |
| 06 | 0 | 0 | 0 | 0 | 0 | 0 | 0 | 5.7x10^4^ | 0 | 0 | 0 | 1.2x10^4^ | 0 | 1.7x10^5^ | 0 | 0 |
| 07 | 0 | 0 | 0 | 0 | 0 | 0 | 4.0x10^5^ | 5.2x10^3^ | 0 | 1.6x10^5^ | 0 | 0 | 0 | 0 | 0 | 0 |
| 08 | 0 | 0 | 0 | 0 | 0 | 0 | 6.5x10^4^ | 0 | 0 | 0 | 0 | 0 | 0 | 8.0x10^3^ | 0 | 0 |
| 09 | 0 | 0 | 0 | 0 | 0 | 0 | 0 | 0 | 0 | 0 | 0 | 2.6x10^3^ | 0 | 1.3x10^5^ | 0 | 0 |
| 10 | 0 | 6.9x10^4^ | 0 | 0 | 0 | 0 | 6.1x10^4^ | 8.1x10^6^ | 0 | 5.7x10^3^ | 0 | 4.4x10^3^ | 0 | 3.0x10^5^ | 0 | 0 |
| 11 | 2.9x10^3^ | 3.4x10^7^ | 0 | 0 | 0 | 0 | 1.1x10^4^ | 4.7x10^3^ | 0 | 2.4x10^3^ | 0 | 0 | 0 | 0 | 0 | 0 |
| 12 | 0 | 0 | 0 | 0 | 0 | 0 | 0 | 0 | 0 | 0 | 0 | 6.6x10^3^ | 0 | 7.4x10^3^ | 0 | 0 |
| 13 | - | 0 | - | 0 | - | 0 | - | 6.2x10^3^ | - | 0 | - | 7.6x10^3^ | - | 2.5x10^5^ | - | 0 |
| 14 | - | 6.4x10^5^ | - | 0 | - | 0 | - | 0 | - | 4.2x10^3^ | - | 1.2x10^5^ | - | 1.0x10^6^ | - | 0 |
| 15 | - | 0 | - | 0 | - | 0 | - | 6.5x10^6^ | - | 1.7x10^5^ | - | 0 | - | 6.1x10^3^ | - | 0 |
| 16 | - | 2.1x10^7^ | - | 0 | - | 0 | - | 1.5x10^7^ | - | 0 | - | 0 | - | 2.0x10^5^ | - | 0 |
| 17 | - | 4.7x10^7^ | - | 0 | - | 0 | - | 1.9x10^7^ | - | 0 | - | 1.7x10^5^ | - | 1.8x10^4^ | - | 7.2x10^3^ |
| 18 | - | 1.1x10^5^ | - | 0 | - | 0 | - | 0 | - | 0 | - | 0 | - | 0 | - | 0 |
| 19 | - | 0 | - | 0 | - | 0 | - | 7.7x10^5^ | - | 0 | - | 4.4x10^4^ | - | 2.7x10^5^ | - | 0 |
| 20 | - | 0 | - | 0 | - | 0 | - | 1.9x10^3^ | - | 0 | - | 1.7x10^4^ | - | 7.8x10^4^ | - | 0 |
| 21 | - | 0 | - | 0 | - | 0 | - | 5.1x10^6^ | - | 2.0x10^4^ | - | 9.0x10^5^ | - | 8.5x10^4^ | - | 0 |
| 22 | - | 0 | - | 0 | - | 0 | - | 6.3x10^3^ | - | 5.6x10^3^ | - | 2.0x10^4^ | - | 9.6x10^4^ | - | 0 |
| 23 | - | 0 | - | 0 | - | 0 | - | 6.6x10^6^ | - | 0 | - | 5.4x10^4^ | - | 6.2x10^4^ | - | 0 |
|  | | | | | | | | | | | | | | | | |

**M^+^ day-14 group**

|  | | | | | | | | | | | | | | | | |
| --- | --- | --- | --- | --- | --- | --- | --- | --- | --- | --- | --- | --- | --- | --- | --- | --- |
|  | **HSV-1** | | **HSV-2** | | **VZV** | | **EBV** | | **CMV** | | **HHV-6** | | **HHV-7** | | **HHV-8** | |
| **Pt.** | **P** | **S** | **P** | **S** | **P** | **S** | **P** | **S** | **P** | **S** | **P** | **S** | **P** | **S** | **P** | **S** |
| 01 | 0 | 0 | 0 | 0 | 0 | 0 | 0 | 3.4x10^4^ | 0 | 0 | 0 | 7.8x10^4^ | 0 | 7.7x10^4^ | 0 | 0 |
| 02 | 0 | 0 | 0 | 0 | 0 | 0 | 0 | 8.0x10^5^ | 0 | 2.4x10^4^ | 0 | 5.3x10^3^ | 0 | 7.1x10^5^ | 0 | 0 |
| 03 | 0 | 0 | 0 | 0 | 0 | 0 | 0 | 3.4x10^3^ | 0 | 4.2x10^3^ | 0 | 1.5x10^4^ | 0 | 7.2x10^4^ | 0 | 6.0x10^3^ |
| 04 | 0 | 4.2x10^3^ | 0 | 0 | 0 | 0 | 0 | 0 | 0 | 0 | 0 | 0 | 0 | 0 | 0 | 0 |
| 05 | 0 | 0 | 0 | 0 | 0 | 0 | 0 | 9.3x10^7^ | 0 | 0 | 0 | 1.9x10^4^ | 0 | 3.1x10^4^ | 0 | 0 |
| 06 | 0 | 0 | 0 | 0 | 0 | 0 | 0 | 5.3x10^6^ | 0 | 0 | 0 | 1.7x10^4^ | 0 | 1.5x10^5^ | 0 | 0 |
| 07 | 0 | 0 | 0 | 0 | 0 | 0 | 1.1x10^4^ | 5.0x10^7^ | 0 | 4.7x10^3^ | 0 | 0 | 0 | 4.3x10^3^ | 0 | 0 |
| 08 | 0 | 0 | 0 | 0 | 0 | 0 | 0 | 1.1x10^6^ | 0 | 0 | 0 | 0 | 0 | 3.3x10^4^ | 0 | 0 |
| 09 | 0 | 0 | 0 | 0 | 0 | 0 | 0 | 0 | 0 | 4.4x10^3^ | 0 | 4.2x10^3^ | 0 | 3.8x10^5^ | 0 | 0 |
| 10 | 0 | 0 | 0 | 0 | 0 | 0 | 0 | 2.4x10^6^ | 0 | 2.9x10^4^ | 0 | 5.0x10^3^ | 0 | 6.6x10^5^ | 0 | 0 |
| 11 | 0 | 2.7x10^4^ | 0 | 0 | 0 | 0 | 0 | 3.0x10^8^ | 0 | 1.2x10^4^ | 0 | 0 | 0 | 5.5x10^2^ | 0 | 0 |
| 12 | 0 | 0 | 0 | 0 | 0 | 0 | 0 | 2.0x10^7^ | 0 | 0 | 0 | 6.7x10^4^ | 0 | 3.1x10^5^ | 0 | 0 |
| 13 | - | 0 | - | 0 | - | 0 | - | 1.1x10^6^ | - | 0 | - | 0 | - | 8.3x10^5^ | - | 0 |
| 14 | - | 0 | - | 0 | - | 0 | - | 2.8x10^6^ | - | 6.4x10^3^ | - | 2.4x10^4^ | - | 5.0x10^5^ | - | 0 |
| 15 | - | 0 | - | 0 | - | 0 | - | 9.5x10^6^ | - | 1.2x10^5^ | - | 1.2x10^3^ | - | 4.1x10^3^ | - | 0 |
| 16 | - | 0 | - | 0 | - | 0 | - | 4.1x10^5^ | - | 0 | - | 0 | - | 3.9x10^4^ | - | 0 |
| 17 | - | 0 | - | 0 | - | 0 | - | 1.0x10^7^ | - | 1.5x10^3^ | - | 6.2x10^4^ | - | 1.6x10^4^ | - | 0 |
| 18 | - | 9.7x10^3^ | - | 0 | - | 0 | - | 0 | - | 0 | - | 1.2x10^4^ | - | 8.4x10^4^ | - | 0 |
| 19 | - | 0 | - | 0 | - | 0 | - | 4.1x10^6^ | - | 0 | - | 1.7x10^5^ | - | 1.6x10^6^ | - | 8.5x10^2^ |
| 20 | - | 0 | - | 0 | - | 0 | - | 2.6x10^4^ | - | 0 | - | 4.4x10^3^ | - | 1.0x10^5^ | - | 0 |
| 21 | - | 0 | - | 0 | - | 0 | - | 6.5x10^6^ | - | 2.3x10^4^ | - | 1.1x10^6^ | - | 4.1x10^4^ | - | 0 |
| 22 | - | 0 | - | 0 | - | 0 | - | 5.0x10^5^ | - | 1.8x10^5^ | - | 3.3x10^4^ | - | 4.6x10^4^ | - | 0 |
| 23 | - | 0 | - | 0 | - | 0 | - | 4.0x10^6^ | - | 0 | - | 1.0x10^5^ | - | 2.3x10^5^ | - | 0 |

**M^-^ group**

|  | | | | | | | | | | | | | | | | |
| --- | --- | --- | --- | --- | --- | --- | --- | --- | --- | --- | --- | --- | --- | --- | --- | --- |
|  | **HSV-1** | | **HSV-2** | | **VZV** | | **EBV** | | **CMV** | | **HHV-6** | | **HHV-7** | | **HHV-8** | |
| **Pt.** | **P** | **S** | **P** | **S** | **P** | **S** | **P** | **S** | **P** | **S** | **P** | **S** | **P** | **S** | **P** | **S** |
| 24 | 0 | 0 | 0 | 0 | 0 | 0 | 0 | 0 | 0 | 0 | 0 | 0 | 0 | 9.0x10^3^ | 0 | 0 |
| 25 | 0 | 0 | 0 | 0 | 0 | 0 | 0 | 0 | 0 | 2.8x10^3^ | 0 | 0 | 0 | 1.3x10^4^ | 0 | 0 |
| 26 | 0 | 0 | 0 | 0 | 0 | 0 | 0 | 1.3x10^5^ | 0 | 0 | 0 | 1.8x10^5^ | 0 | 6.6x10^5^ | 0 | 0 |
| 27 | 0 | 1.5x10^6^ | 0 | 0 | 0 | 0 | 0 | 0 | 0 | 0 | 0 | 0 | 0 | 7.3x10^4^ | 0 | 0 |
| 28 | 0 | 0 | 0 | 0 | 0 | 0 | 0 | 2.0x10^6^ | 0 | 9.3x10^3^ | 0 | 3.3x10^4^ | 0 | 5.1x10^5^ | 0 | 0 |
| 29 | 0 | 0 | 0 | 0 | 0 | 0 | 0 | 0 | 0 | 1.9x10^4^ | 0 | 3.4x10^4^ | 0 | 7.8x10^5^ | 0 | 4.1x10^4^ |
| 30 | 0 | 0 | 0 | 0 | 0 | 0 | 0 | 4.8x10^6^ | 0 | 0 | 0 | 1.1x10^5^ | 0 | 2.9x10^5^ | 0 | 0 |
| 31 | 0 | 0 | 0 | 0 | 0 | 0 | 0 | 2.8x10^6^ | 0 | 9.4x10^1^ | 0 | 4.1x10^3^ | 0 | 4.4x10^4^ | 0 | 0 |
| 32 | 0 | 0 | 0 | 0 | 0 | 0 | 0 | 0 | 0 | 6.5x10^3^ | 0 | 1.3x10^5^ | 0 | 2.8x10^5^ | 0 | 0 |
| 33 | 0 | 0 | 0 | 0 | 0 | 0 | 0 | 5.6x10^5^ | 0 | 8.9x10^4^ | 0 | 3.5x10^4^ | 0 | 4.2x10^5^ | 0 | 0 |
| 34 | 0 | 0 | 0 | 0 | 0 | 0 | 0 | 2.8x10^4^ | 0 | 0 | 0 | 0 | 0 | 3.7x10^4^ | 0 | 0 |
| 35 | 0 | 0 | 0 | 0 | 0 | 0 | 0 | 1.2x10^7^ | 4.4x10^3^ | 7.6x10^5^ | 0 | 1.8x10^5^ | 0 | 1.5x10^6^ | 0 | 0 |
| 36 | - | 1.7x10^8^ | - | 0 | - | 0 | - | 1.8x10^5^ | - | 2.4x10^4^ | - | 1.3x10^5^ | - | 7.2x10^5^ | - | 0 |
| 37 | - | 0 | - | 0 | - | 0 | - | 0 | - | 0 | - | 1.4x10^4^ | - | 2.7x10^4^ | - | 0 |
| 38 | - | 4.7x10^7^ | - | 0 | - | 0 | - | 7.8x10^4^ | - | 2.5x10^4^ | - | 6.1x10^4^ | - | 2.9x10^4^ | - | 0 |
| 39 | - | 0 | - | 0 | - | 0 | - | 0 | - | 4.0x10^4^ | - | 1.1x10^4^ | - | 0 | - | 0 |
| 40 | - | 0 | - | 0 | - | 0 | - | 7.1x10^6^ | - | 0 | - | 0 | - | 2.5x10^3^ | - | 0 |
| 41 | - | 0 | - | 0 | - | 0 | - | 8.1x10^4^ | - | 4.3x10^3^ | - | 3.9x10^3^ | - | 1.0x10^4^ | - | 0 |
| 42 | - | 0 | - | 0 | - | 0 | - | 1.8x10^7^ | - | 0 | - | 1.9x10^4^ | - | 1.9x10^5^ | - | 1.7x10^4^ |
| 43 | - | 0 | - | 0 | - | 0 | - | 0 | - | 0 | - | 0 | - | 3.6x10^4^ | - | 0 |
| 44 | - | 6.4x10^3^ | - | 0 | - | 0 | - | 2.6x10^6^ | - | 0 | - | 3.2x10^3^ | - | 2.0x10^4^ | - | 0 |
| 45 | - | 0 | - | 0 | - | 0 | - | 1.5x10^5^ | - | 1.8x10^4^ | - | 9.9x10^4^ | - | 4.8x10^5^ | - | 0 |
| 46 | - | 0 | - | 0 | - | 0 | - | 5.5x10^4^ | - | 0 | - | 1.2x10^4^ | - | 1.2x10^5^ | - | 0 |
| 47 | - | 0 | - | 0 | - | 0 | - | 0 | - | 0 | - | 1.3x10^6^ | - | 3.5x10^4^ | - | 0 |
|  | | | | | | | | | | | | | | | | |
